# Validation of the Thai short form of the Attitudes to Ageing Questionnaire

**DOI:** 10.1371/journal.pone.0330382

**Published:** 2025-08-18

**Authors:** Nattapat Khongsirisombat, Nareudee Limpuangthip, Pagaporn Pantuwadee Pisarnturakit

**Affiliations:** 1 Faculty of Dentistry, Dental Public Health Program, Chulalongkorn University, Bangkok, Thailand; 2 Department of Prosthodontics, Faculty of Dentistry, Chulalongkorn University, Bangkok, Thailand; 3 Department of Community Dentistry, Faculty of Dentistry, Chulalongkorn University, Bangkok, Thailand; School of Nursing Sao Joao de Deus, Evora University, PORTUGAL

## Abstract

**Introduction:**

The short form of the Attitudes to Ageing Questionnaire was developed to provide a brief, efficient assessment of attitudes toward ageing among older individuals, but requiring less time and energy to administer. However, it has not been translated into Thai language with psychometric validation. This study aimed to validate the psychometric properties of the Thai version of the short form of the questionnaire to determine its reliability in assessing attitudes toward ageing in the Thai context.

**Methods:**

A total of 224 Thai community-dwelling older people aged 60 years or older were included. Participants completed the Thai short form of the Attitudes to Ageing Questionnaire, a 12-item measure with three dimensions: psychological growth, psychosocial loss, and physical change. The questionnaire was translated into Thai using forward and back translation. Psychometric testing comprised confirmatory factor analysis, internal consistency (using Cronbach’s alpha), and test–retest reliability (using the intraclass correlation coefficient).

**Results:**

The results confirmed a three-factor structure corresponding to the original questionnaire’s subscales. Confirmatory factor analysis indicated excellent model fit. Internal consistency was high, with Cronbach’s alpha values of 0.764 for psychological growth, 0.704 for psychosocial loss, and 0.760 for physical change. Test–retest reliability showed excellent stability, with an intraclass correlation coefficient of 0.91.

**Conclusions:**

The Thai short form of the Attitudes to Ageing Questionnaire is a valid, reliable tool for assessing attitudes toward ageing among older Thai individuals. It can assist in identifying areas needing support and informing the development of interventions to promote positive attitudes toward ageing, contributing to the well-being of older adults in Thailand.

## Introduction

The global increase in life expectancy has resulted in there being more older people. Attitudes toward ageing reflect the importance of understanding perceptions of the ageing process. Positive attitudes have been linked to better health outcomes, while negative perceptions can lead to poorer physical and mental health [1–3]. Ageing perceptions are personal evaluations of one’s own ageing process [4], including beliefs, feelings, and attitudes toward one’s own and others’ ageing processes. These perceptions are significant predictors of various health outcomes. Research has indicated that positive perceptions of ageing are associated with increased longevity, improved mental health, and improved quality of life [2,3]. Conversely, negative perceptions can lead to increased stress, depression, and decreased physical health [5,6]. Therefore, measuring attitudes toward ageing is crucial for developing effective policies and interventions to enhance older people’s well-being.

The original Attitudes to Ageing Questionnaire (AAQ) was developed by Laidlaw et al. (2007) as a comprehensive measure of attitudes toward ageing [7]. The AAQ has three subscales: psychological growth (PG), psychosocial loss (PL), and physical change (PC). PG assesses positive experiences and personal growth associated with ageing, such as increased wisdom and life satisfaction. PL assesses negative aspects, such as loneliness, social isolation, and the fear of becoming a burden to others. PC assesses attitudes toward physical health and changes in appearance due to ageing, addressing concerns about declining physical abilities and chronic illnesses [7]. The original AAQ is a self-administered questionnaire with 24 items that assess both positive and negative aspects of ageing, depending on an individual’s experiences and general perceptions of the ageing process [7].

To address the need for a more efficient tool, Laidlaw et al. (2018) developed a short form of the AAQ (AAQ-SF) [8]. The AAQ-SF retains the core elements of the full-length questionnaire, but with fewer items (i.e., 12), making it suitable for large-scale surveys. The AAQ-SF includes the most representative items from each subscale, ensuring that the essential aspects of attitudes toward ageing are still captured effectively. The AAQ-SF has shown strong psychometric properties, including reliability and validity, in various populations [8]. Its brevity allows for quicker administration without compromising the depth of assessment, making it an ideal tool for research and practical applications [8].

The AAQ and AAQ-SF are widely adopted and validated tools used to measure attitudes toward ageing. They have been extensively validated in numerous countries and cultures. Studies have demonstrated the questionnaire’s cross-cultural robustness, establishing it as a valuable tool for international research on attitudes toward ageing [9–14], including the Spanish, Brazilian, Persian, Portuguese, and Malaysian versions [9–13]. The Chinese short form with 12 items has also been validated [14]. These adaptations emphasize the significance of cultural context in how attitudes toward ageing are shaped and underscore the need for culturally sensitive tools.

Thailand, a southeast Asian country, is approaching a completed aged society, where the proportion of people older than 60 years old exceeds 20 percent of the overall population [15,16]. However, there is a lack of validated instruments to measure the attitudes toward and experiences with ageing among older Thai people. Translating and validating the Thai AAQ-SF will address this gap. A Thai AAQ-SF will enable the identification of specific areas wherein older people may need support and will inform the development of targeted interventions to promote positive ageing attitudes in Thailand. We aim to validate the Thai AAQ-SF and examine its psychometric properties, including its reliability and validity. By validating this tool, we will not only contribute to a broader understanding of ageing attitudes in Thailand but also support the development of targeted interventions and policies to improve the well-being of older Thai people worldwide.

## Materials and methods

### Thai translation of the AAQ-SF

Two translators fluent in English and Thai conducted the forward and back translation of the original AAQ-SF into Thai, ensuring the precision of the meaning, intent, and cultural relevance of each item [17,18]. Afterward, the translated Thai questionnaire underwent a thorough review and harmonization to address potential errors, ambiguities, or misinterpretations, following which the final Thai version was agreed upon. Subsequently, the Thai version was independently back translated into English by bilingual translators for comparison and alignment with the original English version, identifying and resolving any ambiguities or discrepancies (S1 File). Once equivalence between the two English versions was achieved, the translated questionnaire underwent pretesting with a small group of older Thai native speakers to validate its comprehensibility and cultural appropriateness (S2 File).

### Participants

Eligible participants were community-dwelling older Thai people aged 60 years or older living in Ang Thong Province, Thailand, without terminal illness or cognitive impairments. Cognitive impairments were ruled out when participants scored less than 4 on the Mini-Cog Test [19], a screening tool used by village health volunteers [20]. Participants were selected through stratified random sampling based on age distribution, with the following age groups: 60–69 years (53%), 70–79 years (31%), and 80 years and older (16%) [21].

To conduct confirmatory factor analysis (CFA), the sample size was determined using an a priori sample size calculator [22] with the following parameters: an anticipated effect size of 0.25, three latent variables, 12 observed variables, statistical power of 0.8, and a probability level of 0.05. A minimum sample size of 181 was calculated, and to compensate for approximately 20% being incomplete data, the sample size was set at 218.

The study was conducted in accordance with the guidelines of the Declaration of Helsinki, and the protocol was approved by the Human Research Committee of the Dental Faculty at Chulalongkorn University (ethical number: HREC_DCU 2023-133). All participants provided written informed consent before data collection. They were informed of the study’s objectives and their right to withdraw at any time without consequences. Data were collected between March 4 and March 8, 2024, through face-to-face interviews conducted by two trained interviewers at participants’ residences.

### Questionnaire interview

Our questionnaire had two parts: 1) sociodemographic characteristics, including sex, age (years), marital status, current living condition, and educational level; 2) the 12-item Thai AAQ-SF. Participants rated each item using a 5-point scale ranging from 1 (*strongly disagree*) to 5 (*strongly agree*). The summed score of each subscale ranged from 4 to 20, but the PL subscale was reverse scored [8]. A higher score suggested a more favorable attitude toward ageing [8].

### Validation procedure and statistical analysis

IBM SPSS Statistics for Windows, Version 29.0 and AMOS, Version 29.0.0 (IBM Corp., Armonk, NY, USA) were used for statistical analysis. The validation procedure and statistical analysis were conducted to assess construct validity through correlation analysis and CFA. Exploratory factor analysis (EFA) was not performed prior to CFA because the AAQ-SF has already been validated in various languages, consistently demonstrating a three-factor structure. Our objective was to confirm the applicability of this established structure within the Thai population, making CFA the clear and effective analytical method for this purpose [23,24]. CFA was performed using AMOS to evaluate model fit, which included a chi-squared test for overall fit and degrees of freedom (*df*). The relative chi-squared (CMIN/DF), root mean square error of approximation (RMSEA), goodness-of-fit index (GFI), and comparative fit index (CFI) values were collected to demonstrate model fit. The Thai AAQ-SF’s reliability was determined with internal consistency using Cronbach’s alpha coefficient and corrected item–total correlations. Additionally, test–retest reliability was assessed by testing 30 participants twice (one week apart) and was reported using the intraclass correlation coefficient (ICC) [25].

## Results

Comparing the Thai AAQ-SF with the original AAQ-SF demonstrated their equivalence. Pretesting the Thai AAQ-SF revealed no issues, indicating that all items were comprehensible and culturally appropriate. Overall, 224 older Thai people with no cognitive issues participated in the interviews with the Thai AAQ-SF, 62.5% of whom were female. The average age of participants was 71.5 years (standard deviation [*SD*] = 8.8), with ages ranging from 60 to 96 years. Their sociodemographic characteristics are shown in Table 1.

**Table 1:** Sociodemographic characteristics of participants.

Characteristic	*N*	Percentage
**Sex**		
Female	140	62.5
Male	84	37.5
**Age group (years)**		
60–69	116	52.0
70–79	60	27.0
80 or older	48	21.0
**Marital status**		
Single	33	14.7
Married	130	58.0
Widowed	47	21.0
Divorced	12	5.4
Separated	2	0.9
**Currently living**		
Alone	19	8.5
With a spouse	32	14.3
With family	173	77.2
**Educational level**		
No education	5	2.2
Elementary education or less	170	75.9
Higher than elementary education	49	23.9

**Fig 1:**
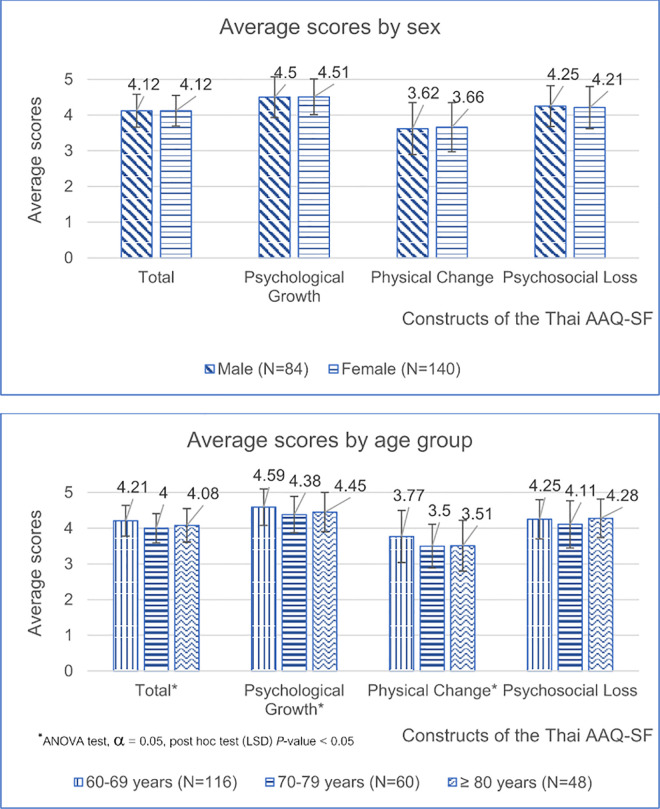
Means and standard deviations of the Thai AAQ-SF by gender and age group. *Chi-squared p-value; alpha = 0.05^θ^; ANOVA p*-*value; alpha = 0.05; post hoc test (LSD).

Analysis of Thai AAQ-SF scores revealed no significant differences between men’s and women’s total and subscale scores (Fig 1). However, significant differences in total mean scores and PG and PC subscale scores were found across age groups.

### Correlation analysis of the Thai AAQ-SF

Extracted factors were constrained to three subscales, aligning with the original 24-item AAQ and the original AAQ-SF. Correlation analysis using Pearson’s correlation coefficient indicated significant correlations among all variables within each subscale (p < 0.01), with correlation coefficients ranging from 0.308 to 0.660 (Table 2). For the PG subscale, the means ranged from 4.440 to 4.650 (*SD *= 0.624–0.721). The highest correlation was observed between Item 8 (passing on the benefits of experiences to younger people) and Item 9 (giving a good example to younger people; 0.660).

**Table 2:** Mean (*x̄*), SD, and Pearson correlation coefficients for the Thai AAQ-SF.

Statement	*x̄*	*SD*	Pearson correlation coefficient			
**Psychological growth**			1	2	8	9
1. It is a privilege to grow old	4.650	0.624	–			
2. There are many pleasant things about growing older	4.480	0.721	0.551*	–		
8. It is very important to pass on the benefits of my experiences to younger people	4.440	0.687	0.329*	0.405*	–	
9. I want to give a good example to younger people	4.450	0.700	0.347*	0.393*	0.660*	–
**Physical change**			4	6	11	12
4. I do not feel old	3.510	0.993	–			
6. I have more energy now than I expected for my age	3.440	0.892	0.497*	–		
11. My health is better than expected for my age	3.570	0.959	0.446*	0.456*	–	
12. I keep myself as fit and active as possible by exercising	4.050	0.845	0.432*	0.361*	0.460*	–
**Psychosocial loss**			3	5	7	10
3. Old age is a depressing time of life	4.340 ^r^	0.629	–			
5. I see old age mainly as a time of loss	4.080 ^r^	0.883	0.325*	–		
7. As I get older, I find it more difficult to make new friends	4.310 ^r^	0.815	0.308*	0.459*	–	
10. I feel excluded from things because of my age	4.160 ^r^	0.840	0.374*	0.389*	0.393*	–

Bartlett’s test of sphericity, χ^2^ (66*, n* = 224) = 758.712, p < 0.001

KMO measure of sampling adequacy = 0.774

*p < 0.01; ^r^ reverse scores

For the PC subscale, the highest correlation was between Item 4 (not feeling old) and Item 6 (having more energy than expected). For the PL subscale, the highest correlation was between Item 5 (seeing old age as a time of loss) and Item 7 (feeling that it is more difficult to make new friends as one gets older). The Kaiser–Meyer–Olkin (KMO) value was 0.774, indicating good sampling adequacy, and Bartlett’s test of sphericity was significant (p < 0.001), confirming data suitability for factor analysis.

### Construct validity

**Fig 2:**
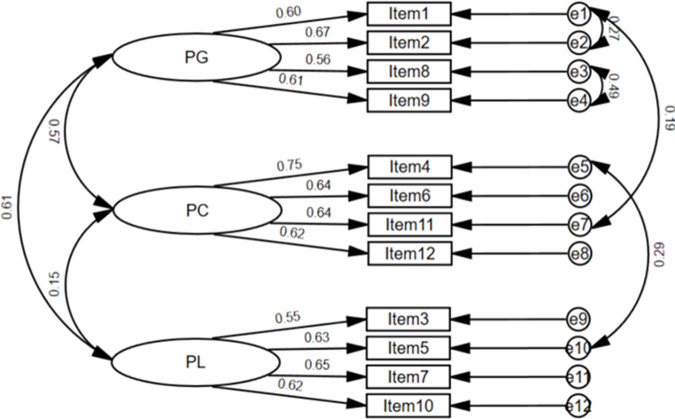
CFA results for the Thai AAQ-SF. Chi-squared = 60.007 (**df* *= 47), p* *= 0.096.

CFA was conducted to validate the identified structure and assess the fit of the model to the collected data. The CFA yielded favorable fit indices (Fig 2). Specifically, the chi-squared test for overall model fit produced a value of 60.007, with *df = *47, indicating a model fit probability greater than 0.05. The CMIN/DF value of 1.277 suggested an acceptable fit [26]. Additionally, the 0.035 RMSEA, 0.957 GFI, and 0.982 CFI values indicated an excellent fit based on the criteria outlined by MacCallum et al. [27], Kline [28], and West et al. [29].

Table 3 presents the standardized factor loadings for each subscale variable of the Thai AAQ-SF. All factor loadings were positive, ranging from 0.557 to 0.667 for the PG subscale, 0.621 to 0.753 for the PC subscale, and 0.547 to 0.647 for the PL subscale, with each loading being statistically significant at p < 0.01. These loadings accounted for approximately 29.9% to 56.8% of the variance. Notably, the variable with the highest factor loading was “I don’t feel old” in the PC subscale, while the lowest factor loading was for “Old age is a depressing time of life” in the PL subscale.

**Table 3:** CFA of the Thai AAQ-SF.

Item and statement	Factor loading			*t*	*R* ^2^
	*b*	SE	β		
**Psychological growth**					
1. It is a privilege to grow old	1.000		0.599		0.359
2. There are many pleasant things about growing older	1.278	0.164	0.668	7.790*	0.446
8. It is very important to pass on the benefits of my experiences to younger people	1.015	0.191	0.557	5.322*	0.310
9. I want to give a good example to younger people	1.138	0.202	0.612	5.639*	0.375
**Physical change**					
4. I do not feel old	1.000		0.753		0.568
6. I have more energy now than I expected for my age	0.758	0.094	0.644	8.049*	0.415
11. My health is better than expected for my age	0.803	0.100	0.638	8.040*	0.408
12. I keep myself as fit and active as possible by exercising	0.693	0.089	0.621	7.826*	0.386
**Psychosocial loss**					
3. Old age is a depressing time of life	1.000		0.547		0.299
5. I see old age mainly as a time of loss	1.617	0.264	0.634	6.122	0.402
7. As I get older, I find it more difficult to make new friends	1.533	0.249	0.647	6.149*	0.419
10. I feel excluded from things because of my age	1.523	0.252	0.624	6.045*	0.389

χ^2^ (1*, n* = 224) = 60.007, *P* = 0.096

*p < 0.01

Fig 2 illustrates the CFA model for the Thai AAQ-SF, which comprised three latent constructs: PG, PC, and PL. Each construct was measured with four observed variables, along with the standardized factor loadings and covariance values between the constructs. The CFA results supported the three-factor structure of the Thai AAQ-SF, with all factor loadings being statistically significant. The model demonstrated an excellent fit to the data, indicating that the Thai AAQ-SF effectively measures the intended constructs related to positive attitudes toward ageing.

### Internal consistency

The Thai AAQ-SF’s reliability was evaluated with internal consistency using Cronbach’s alpha coefficients for the three subscales. The coefficients were 0.760 for PC, 0.704 for PL, and 0.764 for PG, suggesting strong internal consistency among the items in each subscale. Table 4 presents detailed statistics, including scale means and variances if an item was deleted, corrected item–total correlations, squared multiple correlations, and Cronbach’s alpha values if an item was deleted for each item and subscale.

**Table 4:** Item–total statistics and reliability analysis for the subscales of the Thai AAQ-SF.

Item and statement	Scale mean if item deleted	Scale variance if item deleted	Corrected item–total correlation	Squared multiple correlation	Cronbach’s alpha if item deleted
**Psychological growth: Cronbach’s alpha coefficient = 0.764**					
Item 1	13.37	2.915	0.507	0.505	0.737
Item 2	13.54	2.572	0.559	0.58	0.712
Item 8	13.58	2.595	0.596	0.629	0.691
Item 9	13.57	2.56	0.596	0.632	0.691
**Physical change: Cronbach’s alpha coefficient = 0.760**					
Item 4	11.06	4.498	0.583	0.614	0.690
Item 6	11.13	4.942	0.556	0.576	0.705
Item 11	11.01	4.646	0.575	0.604	0.694
Item 12	10.52	5.21	0.522	0.534	0.723
**Psychosocial loss: Cronbach’s alpha coefficient = 0.704**					
Item 3	12.54	3.926	0.43	0.447	0.679
Item 5	12.81	3.017	0.521	0.568	0.622
Item 7	12.57	3.215	0.519	0.561	0.622
Item 10	12.73	3.177	0.505	0.552	0.632

### Test–retest reliability

The Thai AAQ-SF demonstrated excellent test–retest reliability when assessed with the same 30 participants within one week of initial administration. The ICC calculated using a two-way mixed-effects model with absolute agreement and 95% confidence intervals, was 0.91. Notably, values higher than 0.90 indicate excellent reliability [30].

## Discussion

This validation of the Thai AAQ-SF represents a significant advancement in gerontological research within Thailand since we established that this questionnaire is a culturally relevant tool for assessing attitudes toward ageing among older Thai people. The construct validity of the Thai AAQ-SF was confirmed through CFA, from which we identified three factors corresponding to the PG, PL, and PC subscales. This factor structure aligns well with the original AAQ developed by Laidlaw et al. [7]. Additionally, the Thai AAQ-SF demonstrates good reliability, including internal consistency and test–retest reliability. The CFA further validated the model due to the excellent fit indicating that the Thai AAQ-SF is a reliable tool for measuring attitudes toward ageing [7].

Our findings are consistent with those of previous validations of the AAQ in other cultural contexts, such as the Spanish, Brazilian, and Chinese versions [9–14], and in younger age groups [31], wherein robust construct validity was established using similar statistical methods. Similarities between our and other studies’ factor loadings were found. For instance, in the PG subscale, the item on the importance of passing on the benefits of experiences to younger people showed the lowest factor loading, as found in other studies [14,31]. In the PC subscale, the item on keeping oneself as fit and active as possible by exercising showed the lowest factor loading, consistent with another study conducted with those around 50 years of age [31]. In the PL subscale, the item on seeing old age mainly as a time of loss had a high factor loading, similar to what has been found in other studies [14,31,32].

The covariance of the three Thai AAQ-SF subscales revealed that PG and PL had the highest correlation, while PL and PC had the lowest correlation. This correlation trend concurs with the results found among adults in their 50s [31] but differs from findings from China [14]. This difference may be due to the similarities [31] or differences [14] among statements within the subscales.

The Thai AAQ-SF demonstrated strong internal consistency, as shown by high Cronbach’s alpha coefficients for each subscale, indicating that the items within each subscale consistently measure the same underlying construct. The level of internal consistency was similar to the original AAQ’s [7,11,32] and that of its short form [8], which have been validated in various cultural contexts, including French [32] and Chinese [14] populations. The PL subscale had the lowest Cronbach’s alpha coefficient, while the PG and PC subscales were similar across cultural contexts [14,32]. All statements were found to be necessary for each subscale since removing any item would decrease the Cronbach’s alpha coefficient. Specifically, for the item on it being a privilege to grow old, our results indicated that the lowest corrected item–total correlation was in the PG subscale, suggesting that this subscale aligns with the overall scale. Consistent with the findings of Laidlaw et al. [8], this item’s factor loading overlapped with the PC subscale’s, possibly explaining why the item was slightly less consistent with the other items in the PG subscale but was more closely aligned with the PC subscale.

The test–retest reliability of the Thai AAQ-SF was excellent, demonstrating that it provides stable and consistent measurements over time. Previous studies validating the AAQ-SF in other languages, such as Chinese, have also reported similar high test–retest reliability, proving the tool’s reliability across diverse populations [14].

Consistent with previous studies [32], no significant differences were found between men’s and women’s subscale and total scores on the Thai AAQ-SF. Regarding age groups, only the PL subscale showed no differences, possibly because social losses are perceived similarly across age ranges. Contrastingly, the PG and PC subscales appeared to be more directly influenced by age-related changes, which are experienced differently as individuals grow older.

The validation process of the Thai AAQ-SF aligned with previous studies’ findings, reinforcing its applicability in various cultural contexts. For instance, the Spanish validation of the AAQ revealed similar factor structures and high internal consistency, emphasizing the universal features of attitudes toward ageing, despite cultural differences [12]. Validation of the Brazilian version also demonstrated its strong psychometric properties, emphasizing the questionnaire’s effectiveness in capturing ageing attitudes in rapidly ageing societies [9]. These cross-cultural validations indicate that while cultural nuances exist, the core elements of attitudes toward ageing, as measured by the AAQ, are broadly applicable.

We encountered several challenges, particularly due to the varying literacy and comprehension skills among older Thai individuals in rural areas. Consequently, extensive interviews were necessary, and some questions required additional clarifications. Further studies could explore alternative modes of data collection for the Thai AAQ-SF, such as self-administered questionnaires, to enhance accessibility.

Participants in this study were community-dwelling older adults from Ang Thong, Thailand. While data were collected across all districts of the province, most participants were from rural areas. Therefore, caution should be exercised when applying the results to broader contexts. To improve generalizability, the questionnaire should be tested in diverse geographical areas and among different demographic groups, ensuring its relevance to the entire population. In particular, further validation in urban populations and socio-demographically diverse settings is recommended to confirm the tool’s applicability across varied segments of Thai society. The goal is for the Thai AAQ-SF to be widely applicable in both health sciences and social sciences.

## Conclusion

The Thai AAQ-SF was validated, proving that it is a reliable and valid tool for assessing attitudes toward ageing among older Thai individuals. It demonstrates strong construct validity, internal consistency, and test–retest reliability, making it suitable for use in both research and practical settings. The tool can be applied in public health surveillance to monitor perceptions of ageing over time, incorporated into educational programs to raise awareness and foster positive ageing attitudes, and used to inform ageing-related policies and interventions aimed at improving the quality of life for Thailand’s older population.

## Supporting information

S1 File Back-translated English version of the questionnaire. (PDF)

S2 File Thai Short Form of the Attitudes to Ageing Questionnaire; Thai AAQ-SF and English version. (PDF)
