# Supplementary material for: Validation of the Thai short form of the Attitudes to Ageing Questionnaire
Source: PLoS One. 2025 Aug 18;20(8):e0330382. doi: 10.1371/journal.pone.0330382 (PMC12360582; doi:10.1371/journal.pone.0330382)
Supplement: S1 File — (PDF) [file pone.0330382.s001.pdf]

Back-translated English version of the questionnaire

	Consensus Version	Original version	Translator 1	Translator 2
Score	เห็นด้วยอย่างยิ่ง	Strongly agree	Strongly agree	Strongly agree
	เห็นด้วย	Agree	Agree	Agree
	ไม่แน่ใจ	Uncertain	Uncertain	Uncertain
	ไม่เห็นด้วย	Disagree	Disagree	Disagree
	ไม่เห็นด้วยอย่างยิ่ง	Strongly disagree	Strongly disagree	Strongly disagree
**Item**	**Psychological growth**			
1	ฉันรู้สึกโชคดีที่มีชีวิตจนแก่	It is a privilege to grow old	I am lucky to have lived a long life.	I feel so lucky to be alive and getting older and older.
2	มีข้อดีหลายอย่างเมื่ออายุมากขึ้น	There are many pleasant things about growing older	There are many benefits of aging.	There are many pros about being/getting old.
8	การถ่ายทอดประสบการณ์ที่มีประโยชน์ของฉันต่อคนอายุน้อยกว่าเป็นสิ่งที่สำคัญ	It is very important to pass on the benefits of my experiences to younger people	Sharing my valuable experiences to younger generations is important	Passing on my useful experiences for the younger generation is a crucial thing
9	ฉันต้องการเป็นตัวอย่างที่ดีให้กับคนที่อายุน้อยกว่า	I want to give a good example to younger people	I want to set a good example for younger people.	I want to be a good role model for the younger generation
**Item**	**Physical change**			
4	ฉันไม่รู้สึกว่าตัวเองแก่	I don’t feel old	I don't feel old.	I don't feel that that I’m old.
6	ในวัยของฉัน ฉันมีแรงมากกว่าที่คิดไว้	I have more energy than I expected for my age	At my age, I have more strength than I anticipated.	At my age, I’m healthier/stronger than I thought
11	ในวัยของฉัน ฉันมีสุขภาพดีมากกว่าที่คิดไว้	My health is better than expected for my age	At my age, I am more healthy than I anticipated.	At my age, I’m in good shape/healthier than I thought.
12	ฉันดูแลตัวเองให้แข็งแรงและกระฉับกระเฉงเท่าที่จะเป็นไปได้ด้วยการออกกำลังกาย	I keep myself as fit and active as possible by exercising.	I keep myself healthy and active as possible, through exercise.	I take care of myself and stay active as much as I can by exercising.
**Item**	**Psychosocial Loss**			
3	วัยชราเป็นช่วงเวลาที่น่าหดหู่ของชีวิต	Old age is a depressing time of life.	Old age is the most depressing period of life	Senility is the period of depression/gloom/ distress.
5	ฉันเห็นว่าวัยชราเป็นช่วงเวลาของการสูญเสีย	I see old age mainly as a time of loss.	Aging can be seen as a succession of losses.	I think aging/senility/geriatric is about the time of loss.
7	ฉันหาเพื่อนใหม่ได้ยากขึ้นเมื่อมีอายุมากขึ้น	As I get older, I find it more difficult to make new friends.	Aging make making friend harder.	I found it hard to make new friends after getting older.
10	ฉันรู้สึกถูกกีดกันออกจากสิ่งต่างๆเมื่ออายุมากขึ้น	I feel excluded from things because of my age.	I feel like I am excluded out of many things, as I age.	As I get older, I feel left out.
