# Supplementary material for: Validation of the Thai short form of the Attitudes to Ageing Questionnaire
Source: PLoS One. 2025 Aug 18;20(8):e0330382. doi: 10.1371/journal.pone.0330382 (PMC12360582; doi:10.1371/journal.pone.0330382)
Supplement: S2 File — (PDF) [file pone.0330382.s002.pdf]

แบบประเมินทัศนคติต่อการสูงอายุแบบย่อภาษาไทย

(Thai Short Form of the Attitudes to Ageing Questionnaire; Thai AAQ-SF)

คำแนะนำ คำถามต่อไปนี้ถามว่าคุณเห็นด้วยกับข้อความต่อไปนี้มากน้อยเพียงใด กรุณาตอบคำถามทุกข้อ

ข้อ	คำถาม	เห็นด้วยอย่างยิ่ง	เห็นด้วย	ไม่แน่ใจ	ไม่เห็นด้วย	ไม่เห็นด้วยอย่างยิ่ง
1	ฉันรู้สึกโชคดีที่มีชีวิตจนแก่	5	4	3	2	1
2	มีข้อดีหลายอย่างเมื่ออายุมากขึ้น	5	4	3	2	1
3	วัยชราเป็นช่วงเวลาที่น่าหดหู่ของชีวิต	5	4	3	2	1
4	ฉันไม่รู้สึกว่าตัวเองแก่	5	4	3	2	1
5	ฉันเห็นว่าวัยชราเป็นช่วงเวลาของการสูญเสีย	5	4	3	2	1
6	ในวัยของฉัน ฉันมีแรงมากกว่าที่คิดไว้	5	4	3	2	1
7	ฉันหาเพื่อนใหม่ได้ยากขึ้นเมื่อมีอายุมากขึ้น	5	4	3	2	1
8	การถ่ายทอดประสบการณ์ที่มีประโยชน์ของฉันต่อคนอายุน้อยกว่าเป็นสิ่งที่สำคัญ	5	4	3	2	1
9	ฉันต้องการเป็นตัวอย่างที่ดีให้กับคนที่อายุน้อยกว่า	5	4	3	2	1
10	ฉันรู้สึกถูกกีดกันออกจากสิ่งต่างๆเมื่ออายุมากขึ้น	5	4	3	2	1
11	ในวัยของฉัน ฉันมีสุขภาพดีมากกว่าที่คิดไว้	5	4	3	2	1
12	ฉันดูแลตัวเองให้แข็งแรงและกระฉับกระเฉงเท่าที่จะเป็นไปได้ด้วยการออกกำลังกาย	5	4	3	2	1

The Short Form of the Attitudes to Ageing Questionnaire (AAQ-SF)

Instructions: The following questions ask how much you agree with each statement. Please answer all items.

Item	Question	Strongly agree	Agree	Uncertain	Disagree	Strongly disagree
1	It is a privilege to grow old	5	4	3	2	1
2	There are many pleasant things about growing older	5	4	3	2	1
3	Old age is a depressing time of life.	5	4	3	2	1
4	I don't feel old	5	4	3	2	1
5	I see old age mainly as a time of loss.	5	4	3	2	1
6	I have more energy than I expected for my age	5	4	3	2	1
7	I see old age mainly as a time of loss.	5	4	3	2	1
8	It is very important to pass on the benefits of my experiences to younger people	5	4	3	2	1
9	I want to give a good example to younger people	5	4	3	2	1
10	I feel excluded from things because of my age.	5	4	3	2	1
11	My health is better than expected for my age	5	4	3	2	1
12	I keep myself as fit and active as possible by exercising.	5	4	3	2	1
